# Assessing the carbon capture potential of a reforestation project

**DOI:** 10.1038/s41598-021-99395-6

**Published:** 2021-10-07

**Authors:** David Lefebvre, Adrian G. Williams, Guy J. D. Kirk, Paul, J. Burgess, Jeroen Meersmans, Miles R. Silman, Francisco Román-Dañobeytia, Jhon Farfan, Pete Smith

**Affiliations:** 1 School of Water, Energy and Environment, Cranfield University, Cranfield, MK43 0AL Bedfordshire UK; 2 TERRA Teaching and Research Centre, Gembloux Agro-Bio Tech, University of Liège, 5030 Gembloux, Belgium; 3 Centro de Innovación Científica Amazónica-CINCIA, 17001, Madre de Dios, Peru; 4 Center for Energy, Environment and Sustainability, Wake Forest University, Winston-Salem, NC 27106 USA; 5 Department of Biology, Wake Forest University, Winston-Salem, NC 27106 USA; 6 Institute of Biological and Environmental Sciences, University of Aberdeen, 23 St Machar Drive, Aberdeen, AB24 3UU UK

**Keywords:** Climate-change mitigation, Environmental impact

## Abstract

The number of reforestation projects worldwide is increasing. In many cases funding is obtained through the claimed carbon capture of the trees, presented as immediate and durable, whereas reforested plots need time and maintenance to realise their carbon capture potential. Further, claims usually overlook the environmental costs of natural or anthropogenic disturbances during the forest’s lifetime, and greenhouse gas (GHG) emissions associated with the reforestation are not allowed for. This study uses life cycle assessment to quantify the carbon footprint of setting up a reforestation plot in the Peruvian Amazon. In parallel, we combine a soil carbon model with an above- and below-ground plant carbon model to predict the increase in carbon stocks after planting. We compare our results with the carbon capture claims made by a reforestation platform. Our results show major errors in carbon accounting in reforestation projects if they (1) ignore the time needed for trees to reach their carbon capture potential; (2) ignore the GHG emissions involved in setting up a plot; (3) report the carbon capture potential per tree planted, thereby ignoring limitations at the forest ecosystem level; or (4) under-estimate tree losses due to inevitable human and climatic disturbances. Further, we show that applications of biochar during reforestation can partially compensate for project emissions.

## Introduction

Carbon (C) sequestration programs are necessary to reach the UNFCCC Paris Agreement targets and limit the global average temperature increase to well below 2 °C^1^. Planting trees is an effective way to capture C^2^. Compared with other greenhouse gas (GHG) capture practices, it is cheap and easy to set up using established technology^3^. The number of tree planting projects globally has increased in the past decade^4^, with the aim of both supporting livelihoods and sequestering carbon dioxide (CO_2_) into long-term biomass^1,5^. This year marks the beginning of the United Nation decade on ecosystem restoration where incentives will be put in place to restore degraded ecosystems, in part through reforestations^6^. However, the extent to which such projects can contribute to global GHG capture targets is debated^7^ and it is important that claimed sequestration potentials are realistic.

So-called reforestation platforms distribute funding between different reforestation/afforestation projects. Reforestation platforms are responsible for planting large numbers of trees. *Ecosia,* for example, has planted over 100 million trees in more than 25 countries since its creation in 2009^8^. Reforestation platforms often cite a C or CO_2_ capture figure per tree planted^9,10^, rather than at the forest ecosystem level. Carbon sequestration claims are often calculated ahead of time based on expected wood density and maximum height of the planted trees^10^, following carbon removal rates from published studies (e.g. Bernal et al.^11^), or they are sometimes left to certification bodies, using similar techniques (e.g. Gold Standard^12^, Verra^13^). This ignores the fact that reforestation is a long term undertaking and failure rates are often high, for example where a lack of soil care and seedling protection results in tree death during establishment^4,14^. In addition, many steps are involved in the setup and maintenance of a reforestation plot, all involving some type of energy consumption, leading to GHG emissions^15^. However, information from reforestation platforms about the time needed for capture claims to be realised, or the environmental impact of the reforestation itself, are often over-looked^9,16,17^.

**Figure 1:**
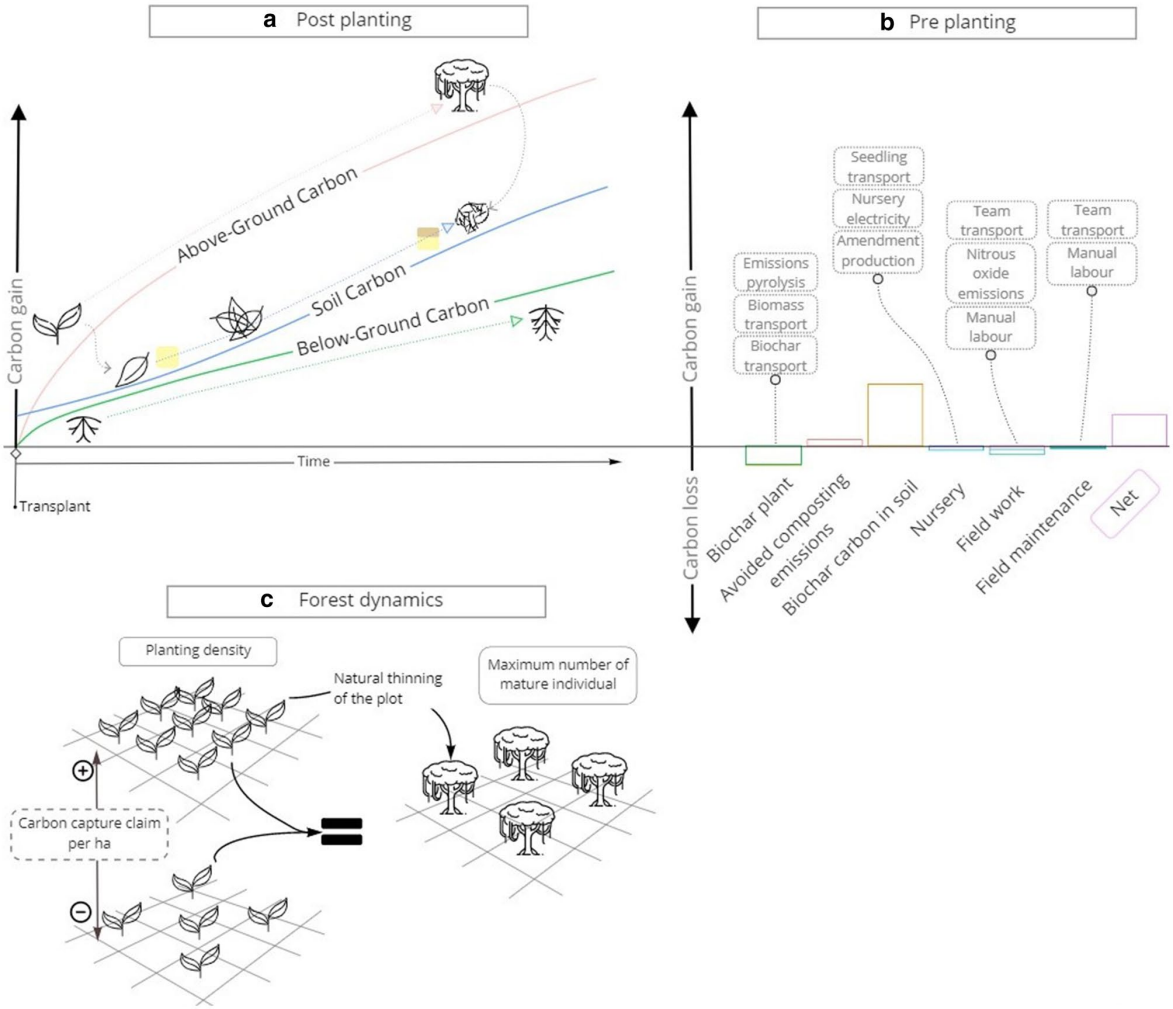
Descriptive schematic figure of the processes and stocks considered. (**a**) Vegetation and soil C stocks. (**b**) Processes included in the LCA and their relative impact. c effect of the planting density.

In this study, we use life cycle assessment (LCA) to empirically derive the carbon footprint of setting up and maintaining a reforestation plot for one year in a tropical forest (“Methods”). We include a focus on biochar, a recently adopted soil amendment of widespread interest, used in the plot studied^18^. In parallel, we combine above and below ground biomass models with a soil carbon model, RothC^19^, to provide an evolving carbon capture profile tailored for the tropical plot under study. We then compare our results with claims made by a typical reforestation platform and discuss their relevance. Informative representation of the processes and stocks accounted for in this study is shown in Fig. 1.

## Methods

### Study area

**Figure 2:**
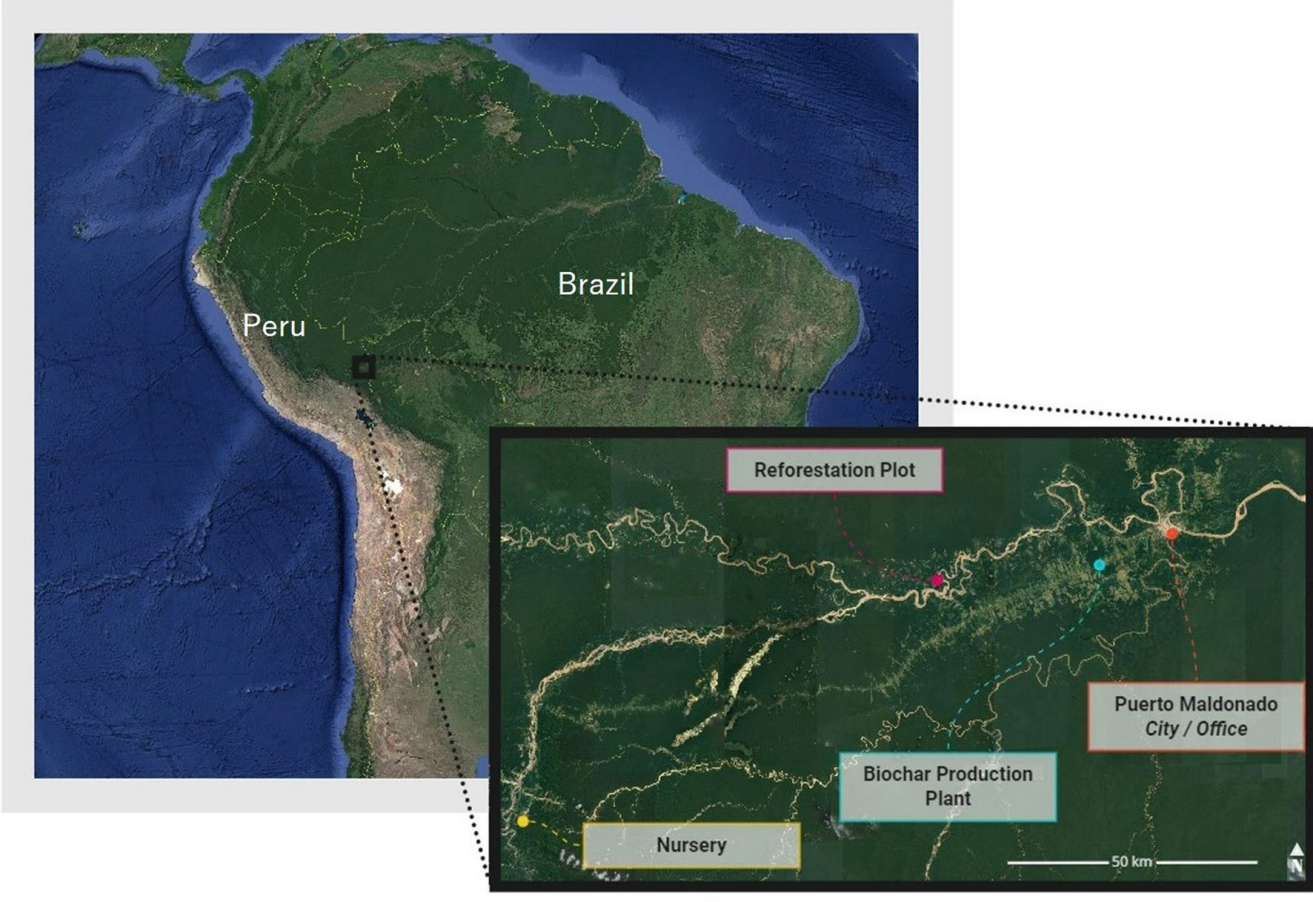
Case study location. Generated using Google Earth Pro (version 7.3.4.8248)^22^.

The present LCA is based on a reforestation project set up and maintained by the Centre for Amazonian Scientific Innovation (CINCIA^20^), in the Peruvian Amazon. CINCIA has, so far, reforested 42.5 ha of degraded forest using 74 tree species on 19 different plots^21^. The plot studied here is located in the south-eastern Peruvian Amazon region of Madre de Dios (12° 41′ 3.15″ S, 69° 36′ 47.76″ W) at an altitude of 266 m above sea level (Fig. 2). It corresponds to Site 1 in Román-Dañobeytia et al.^18^ and is on an open sandy area under an enriched biochar treatment including biochar and fertilizer (“Supplementary Information”)^18^.

### System boundary and functional unit

**Figure 3:**
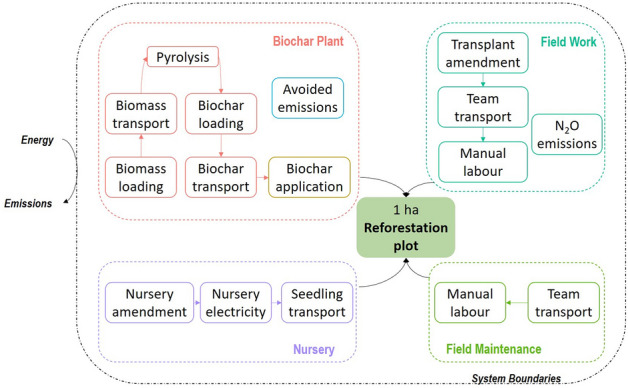
System boundaries of the study.

The carbon footprint of this case study includes processes from the receipt of the seeds by the nursery, their development into seedlings and transport to the reforestation plot. In parallel, the system boundaries include the biomass collection, its transformation into biochar and the transportation of the amendments to the reforestation plot. They also include the field work necessary at the time of transplant and for the maintenance of the seedling up to one year after transplant (Fig. 3), following which, no further care of the plot is made by the team. The reforestation takes place in a protected area and hence the LCA excludes timber collection and assumes that the area is left untouched indefinitely. We use 100-year global warming potentials (GWP) as prescribed in the IPCC Fifth Assessment Report (AR5)^23^ and “one reforested hectare” as the functional unit.

### Software, database and data processing

Emissions factors of processes and fuels were assessed using EcoInvent 3.7 and USLCI databases in SimaPro 8.3^24^ and their carbon footprint calculated using the IPCC 2013 GWP 100a V1.03 method^25^. Data and emissions factors are available in the “Supplementary Information”. Life cycle impact assessment, uncertainty analysis (Monte Carlo method), modelling activities, and figures were produced using R software (version 3.5.1)^26^.

### Biochar

Biochar is produced and added to the site at the time of seedlings transplant at a rate of one kg (dry mass) per seedling^18^. The biochar is produced using Brazil nut husks, residues of the local Brazil nut production^27^. Pyrolysis of Brazil nut husks avoids the emissions associated with piling these residues in unmanaged heaps in the environment, as is it commonly done in the region (Jhon Farfan, *pers. com*.). First, the husks are loaded in a medium scale truck using a front loader. The truck then drives an average distance of 20 km from the city to the biochar producing area and unloads the biomass. The biomass is then sun-dried and manually loaded into a top-lit up-draft (TLUD) biochar pyrolyzer^27^. It is then loaded into a truck and a boat, to be transported to the plot and applied alongside each seedling in the planting hole.

### Nursery

CINCIA possesses its own technological tree nursery where the seeds develop into seedlings before transplant^28^. The seedlings are grown in reusable plastic nursery tubes in an in-house growing medium (“Supplementary Information”). CINCIA’s seedling transplant rate is 1111 seedlings ha^−1^^18^. The nursery grows seedlings in batches and requires 20.5 kWh electricity per month (“Supplementary Information”). The seedlings need 5 months to reach a sufficient height (Jhon Farfan, *pers. com*.), hence the average electricity needed reaches 102 kWh per hectare. The seedlings are then transported from the nursery to the field by truck (128 km) and boat (9 km) to reach the plot.

### Field work

At transplanting, each seedling is planted along with 1 kg biochar followed by a surface application of an additional 100 g granulated N–P_2_O_5_–K_2_O 20–20–20^18^. All field work is done manually and requires eight people per hectare for around 3 days (Jhon Farfan, *pers. com*). We accounted for the emissions associated with manual labour according to Rugani et al.^29^ considering the purchasing power parity of Peru^30^ for increased accuracy. The field work process also accounts for the direct and indirect N_2_O emissions associated with the nitrogen content of all amendments following the Tier 1 method^31^.

### Field maintenance

Field maintenance (cleaning) is required three times in the first year (at 3, 6, and 12 months)^28^. We accounted for a team of eight people per hectare working for eight hours for each maintenance round. Manual labour was accounted for similarly to field work.

### Soil carbon modelling

We used the RothC soil carbon model^19^ to simulate changes in the soil carbon stock over time after transplanting. We used RothC in inverse mode to get insights into the total carbon input needed to maintain the soil carbon stock at the level of the degraded areas and the adjacent forest. We then computed a sigmoid curve between the forest litter input needed for the degraded soil C stock (minimum litter input) and the maximum forest litter input needed to reach the adjacent forest soil C stock (maximum litter input) to describe the required carbon input over time, reaching maximum carbon input 40 years after transplanting^32,33^ and used this increasing carbon input over time in RothC to compute the soil carbon change following seedling transplant (as described in Cerri et al.^34^). We accounted for biochar soil carbon stock impacts following the approach described in Lefebvre et al.^35^ and used their modified coding of RothC in R. Meteorological data, soil and additional data required for RothC are given in the “Supplementary Information” file.

### Above- and below-ground carbon modelling

**Figure 4:**
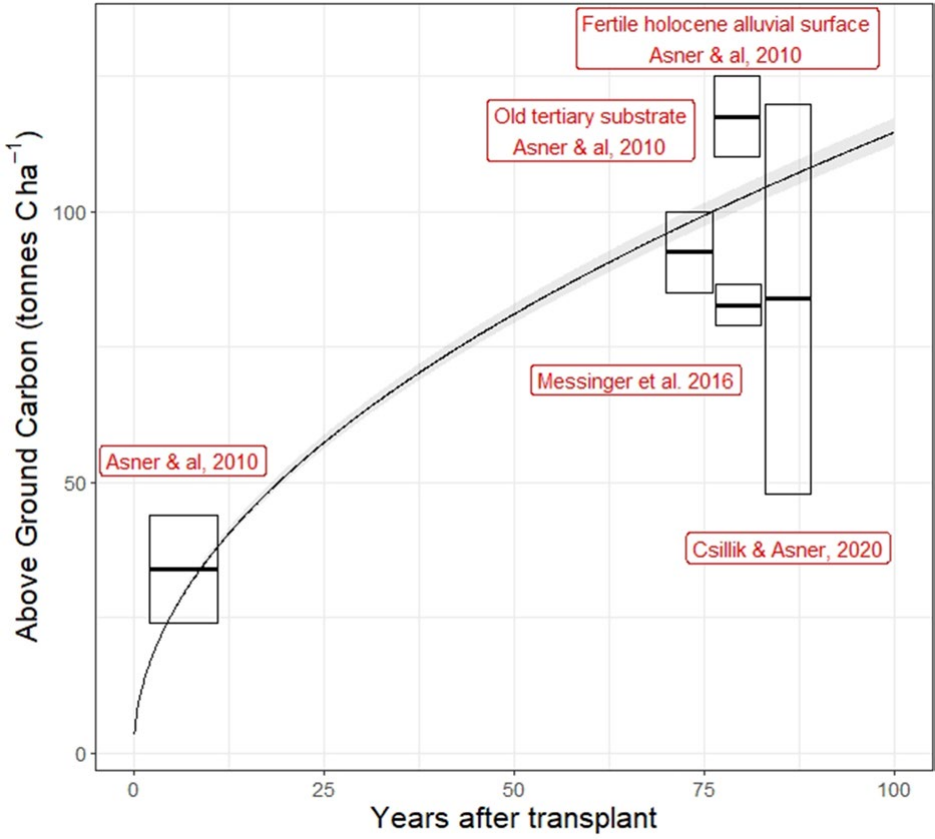
Above-ground carbon model (black line) and boxes representing above-ground carbon values from the literature of forest plot nearby our case study. Forest plots represented by the boxes on the right hand side were assumed to be a mature forest (≈ 80 years)^2,33,41,42^.

For the above-ground carbon model we used the moist forest, non-plantation model reported in Busch et al.^36^, which simulates tropical secondary forests above-ground growth based on data from 829 tropical forest stands. We tested the model by comparing it with above-ground carbon value measured on plots near the case study plot^37–39^ (Fig. 4). The model values are in the lower range of above-ground carbon stock increase for Peruvian forests^40^.

The below-ground carbon model has an evolving root to shoot ratio over time, specific for tropical forests, with the root to shoot ratio of one tree increasing with tree size (ranging from 0.29 to 0.65 for young and old growth forests, respectively)^43^.

### Comparison with a reforestation platform

First, we used our three C pool model to foresee the time needed to reach a claim of 100 kg CO_2_ captured tree^−1^ planted (consistent with existing reforestation platforms^9,10,16^) considering the planting density similar to the one presented in the case study (1111 seedling ha^−1^). Similarly, we assumed the emissions associated with the nursery, field work and field maintenance processes equivalent to our case study. In addition, we considered that reforestation platform represented here does not use biochar and only reforests on highly degraded soils as the one type in our case study. Then we modify the planting density and observe its impact on the time needed to reach the carbon capture claim. Data used for the modelling activities is available in the “Supplementary Information”.

## Results

### Modelling soil and biomass pools

**Figure 5:**
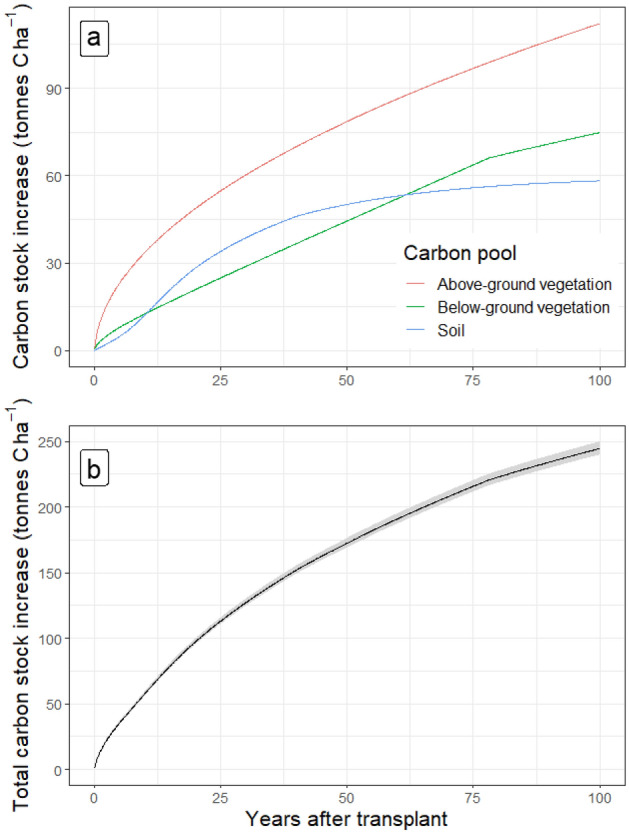
Changes in carbon stocks following transplanting. (**a**) Vegetation and soil. (**b**) Total, with ± 1 SD indicated by the ribbon.

The C content of topsoil took around 50 years to reach steady state following transplanting the new forest, while both above- and below-ground vegetation pools increased over 100 years at a declining rate over time (Fig. 5a)^32,44^. Simulations of the below-ground biomass model matched a previous assessment^45^ and the evolution of total carbon stocks after transplant (Fig. 5b) is consistent with previous assessments^32,46^. About 80 years after transplanting, the additional carbon stocks comprise 45% above-ground carbon, 30% below-ground carbon and 25% soil carbon.

### Life cycle assessment of the case study plot

**Figure 6:**
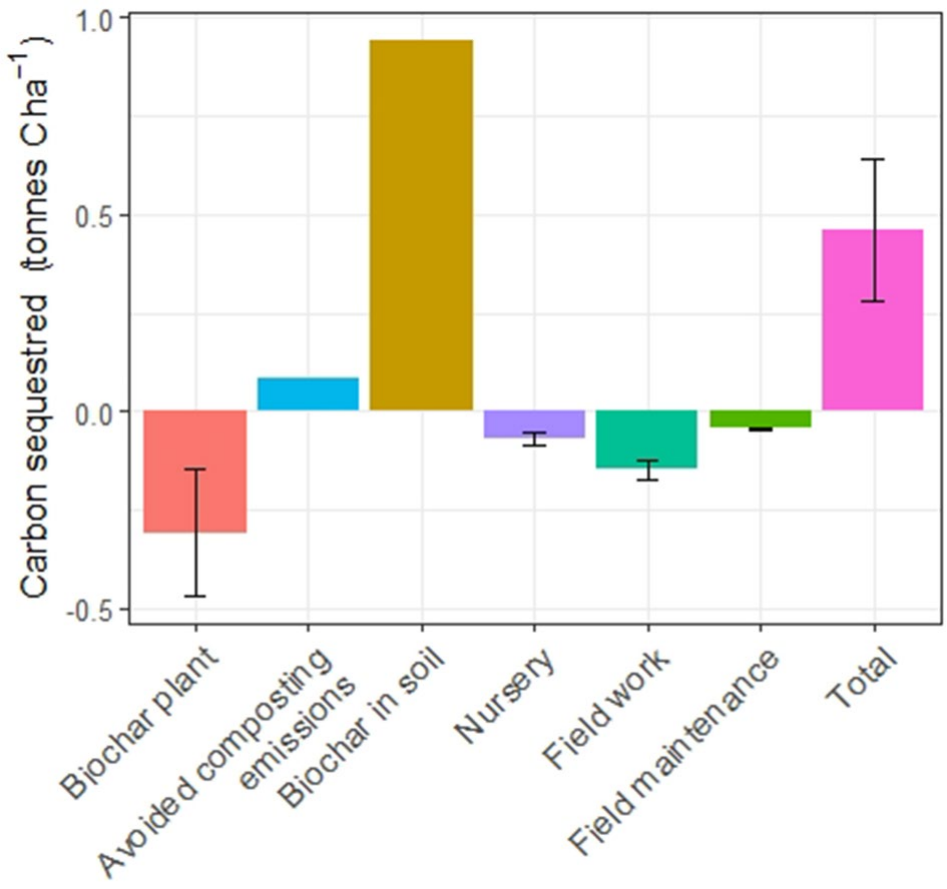
Grouped contribution analysis of the emission and emission reduction associated with reforesting one hectare from our case study plot including one year maintenance. The error bars represent ± 1 SD.

The LCA shows that using biochar as a reforestation amendment delivered a net capture of 1.87 ± 0.66 t CO_2_e ha^−1^ (0.51 ± 0.18 t C ha^−1^) within the first year after planting (Fig. 6), but the biochar opportunity was site specific. Most of the establishment emissions arise from pyrolysis, fertilizer manufacture and use, and associated N_2_O emissions, and 90% of the sequestration comes from the 1.11 t biochar ha^−1^ application (capturing 3.45 t CO_2_e ha^−1^, i.e. 0.94 t C ha^−1^). Excluding biochar production and use, establishing the case study plot emits 1.27 ± 0.10 t CO_2_e ha^−1^ (0.35 t C ha^−1^). The complete contribution analysis is given in the “Supplementary Information” file.

### Comparison with a hypothetical reforestation hub

**Figure 7:**
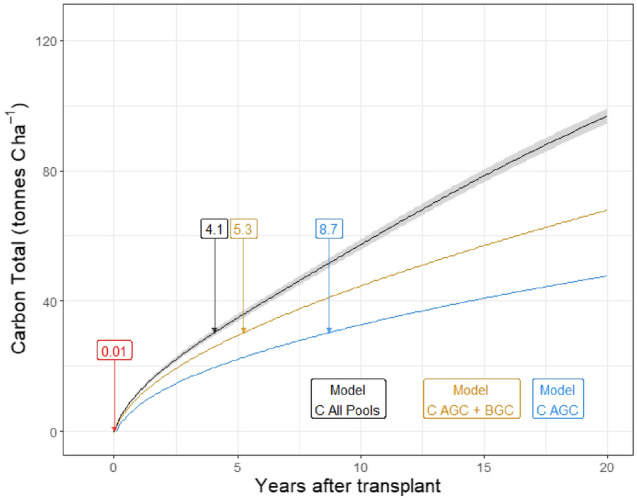
Time needed in years (labels) to reach estimated claim of 100 kg CO_2_e captured per tree using our model considering all C pools (black), above and below-ground C pool only (yellow), and above-ground C pool only (blue). The red label represents the time in years necessary to offset the emissions associated to set up the plot. The ribbon around the model carbon capture rate is ± 1 SD.

We calculate it would take 4.1 years after transplanting to capture 100 kg CO_2_ per tree planted in our case study plot (including all C pools—Fig. 7), which is a typical reforestation platform target. With a planting density of 1111 seedlings per hectare (3 m spaced grid) 100 kg CO_2_ (i.e., 27.3 kg of C) per tree equates to 111,100 kg CO_2_ (i.e., 30,300 kg of C) captured per ha after 4.1 years. Without biochar, setting up the case study reforestation plot would emit 1.27 ± 0.10 t CO_2_e ha^−1^. This carbon debt is quickly covered by the growing seedlings (Fig. 7). If we only account for aboveground biomass (i.e., excluding soil C stock and below-ground C) our model predicts 8.7 years to reach the 100 kg CO_2_ carbon capture target per tree.

**Figure 8:**
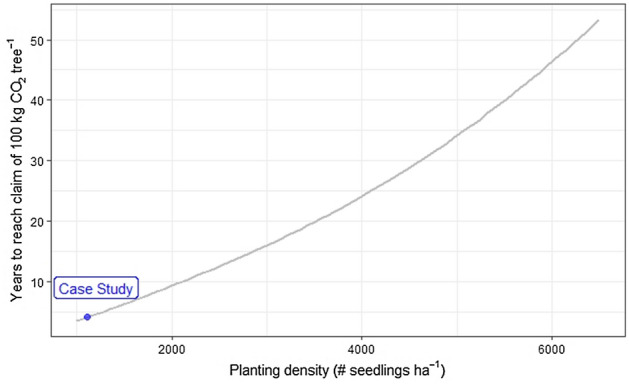
Effect of the planting density on the plot-scale carbon capture rate. No manual thinning and 100% seedling transplant survival are assumed. The blue dot represents the planting density of 1111 seedling ha^−1^ (where our model reach 100 kg CO_2_ captured per tree 4.1 years after planting).

Figure 8 shows the time needed to reach the 100 kg CO_2_ tree^−1^ as a function of seedling density. Following the carbon capture claim per tree, the more numerous the seedlings planted per hectare, the higher the total carbon claim per hectare. However, considering the non-linear increase in C stocks in a growing forest (Fig. 5), the higher the total carbon capture claim per hectare, the longer it will take for the plot to achieve the claim.

## Discussion

Our study shows that the case-study plot would need to be cared for and maintained for 4.1 years before the 100 kg CO_2_e captured per tree claimed could be reached (considering 1111 seedling ha^−1^). These modelled numbers are likely to be at the lower-end of actual values achieved in the field as they were derived on a soil that was highly degraded and the carbon stock consequently increased by around 40 t C ha^−1^ in the first 50 years (Fig. 5). In addition, the biomass growth is modelled for low altitude tropical rainforest, which is not representative of all reforestation projects. Whereas the carbon sequestration occurs in the future, some reforestation platforms assume the sequestration is immediate^9,17^. This potentially misleads supporters and buyers into thinking that their carbon offsets are both instantaneous and permanent. Despite the importance of these reforestation platforms for climate change mitigation, their forward carbon capture claims can be misinterpreted due to (1) the omission of forest growth trajectory, (2) by ignoring the emissions associated with the reforestation and monitoring processes, and (3) the risk that either the trees die or be destroyed or that they are harvested for a use where the carbon is soon released as carbon dioxide^47^. These amplified and premature C capture values can allow companies to claim substantial carbon footprint reductions by massively investing in reforestation projects (e.g. Shell^48^), hence misleading the public on their actual impact. This practice also risks devaluing CO_2_ credits which, in turn, could undermine the attractiveness of other carbon capture techniques or platforms.

We show that GHG emissions associated with establishing the plot need to be factored into the carbon accounting. The amendments and substrates needed in the nursery and their associated nitrous oxide emissions represent a substantial share of the contribution analysis (“Supplementary Information”). As a result, planting density and seedling mortality should be monitored and minimised to reduce the overall carbon footprint of establishing the plot. Excluding biochar production and use, establishing the case study plot emitted 1.27 t CO_2_ ha^−1^, which is very similar to reforesting open woodland in a Canadian boreal forest, i.e. 1.25 t CO_2_ ha^−1^^15^. Although low and being rapidly offset by the growing forest (Fig. 7), projects relying on heavy machinery for land preparation, transporting and spreading fertile soil (the practice of “re-soiling”, sometimes used for land reclamation/restoration^49^), eliminating competitive shrubs, mounding or subsoiling the plot to increase seedling growth and/or survival^50^ will have a higher establishment carbon footprint to offset before any carbon capture claim can be made. For instance, subsoiling would increase the carbon debt by an average of 41.4 ± 10.3 kg CO_2_e ha^−1^^51^ (apart from transporting the machinery to the site).

The use of biochar provides an opportunity to offset the GHG emissions associated with establishing the plot. Using another biochar feedstock that is not destined to be composted, but could be used for other purposes, would reduce the overall capture of the practice by at least 8% (solely accounting for the avoided composting emissions—“Supplementary Information”). On the other hand, improving the pyrolysis process by improving equipment performance (at an economic cost) and reducing its emissions by half, would increase the carbon capture potential of using biochar by 30%. Similarly, applying the biochar at the hectare scale instead of locally to each seedling would vastly increase the carbon sequestration.

We used an above-ground carbon model of secondary tropical forest growth. Natural forest regeneration models do not accurately represent the reforestation process where seedlings are deliberately transplanted to increase cover and forest regeneration. But they do account for the natural thinning of the plot as trees mature, which is not replicated by an individual seedling’s growth trajectory on recently reforested land. In addition, studies show that natural regeneration can be more effective for increasing carbon stocks than reforestation, depending on the former land use and location of the site^52,53^. The speed at which carbon stocks increase in the natural regeneration model is not altered by the planting density of the hypothetical reforestation project it represents. The asymptotic shape of the calculated change in above-ground C (Fig. 5a) symbolises a rapid colonization of the plot by pioneer species followed by a slow reduction in rate over time as the number of individuals thins out and the respiration load of the woodland increases. Reforestation platforms’ C capture claims are based on individual trees, meaning that claims per hectare are highly dependent on the planting density. Figure 8 shows that planting density clearly affects the time needed to reach the carbon capture claim per tree. Nevertheless, no matter what the original planting density was, the maximum number of thriving individual trees per hectare is limited by environmental parameters^54^. Thinning is seen in the natural regeneration model used in this study (characterized by its asymptotic shape). Although advertising the carbon capture impact of each tree planted may be an effective communication strategy by reforestation platforms, these observations should motivate carbon capture verification bodies to require carbon capture claims per hectare using context-specific models accounting for ecosystem limitations, ideally supplemented by on-site surveys. A major issue when foreseeing carbon capture figures at the individual tree level is that it implies that all trees will prosper and reach maturity, which overlooks the competition between individual trees for light and other resources leading inevitably to a maximum number of individuals of a given size that any specific area can sustain^54^.

Growing forests are subject to numerous threats. The frequency of wildfire has increased in rainforests over the past decades^55^. While it is agreed that wildfires have a major negative impact on forest carbon stocks^56^, accounting for all potential effects of the fire on the regrowth of a forest plot is difficult^55^. Although wildfires happen predominantly on old growth forest, with more dead material in old growth forests compared to secondary forests^57^, tree mortality has increased in young secondary forests, particularly in the western and southern Amazon^58^. In addition, droughts have also become more frequent in Amazonia leading to potential lasting degradation of these ecosystems and their carbon sink ability^59,60^. The increase in atmospheric CO_2_ concentration can increase tree growth in the absence of other constraints^61^ but faster growth can lead to reduced tree lifespans with a negative impact on total carbon sequestration capacity^62^. These observations add to the overall adverse effects of climate change on the C sink of the Amazon^60,61^. While the prospective impacts of wildfires, droughts or climate change are difficult to assess for a recently reforested plot in a remote location of the rainforest, their occurrence and impact have increased. Further, diseases can have a disastrous impact on the survival of some tree species^63^. Hence, there is a substantial risk in assuming that an unsupervised young reforested plot, particularly of a single species, will provide permanent carbon sequestration.

Brander et al. argue that businesses should only report CO_2_ capture figures that have been already and permanently captured^64^. An attraction of carbon storage in the above-ground biomass of forests is that it can be readily assessed using remote sensing techniques supported by surveys^65^. However, as discussed earlier, the above-ground C credits associated to the future growth of reforestation projects come with associated risks, and the figures should be frequently monitored and evaluated to ensure appropriate accuracy^64,66^. Another solution would be to set up a carbon stock discount rate of newly planted forests based on the risks of plot destruction. A newly planted forest could be applied a discount rate on its C capture potential based on the socio-political status or environmental disaster potential of the area in which it is planted. This discount rate would need to be context specific and agreed upon. Ex-post calculation of the C internal rate of return of the project (including emissions to set up the plot) and comparison with the local discount rate could provide insight on whether or not the plot is worth setting up. Some authors argue that the evolution of soil carbon stocks in reforested land can be a more durable form of carbon stock less prone to disturbance (e.g. logging or fire) than the above-ground stock^46^ and reach equilibrium faster, at least in the tropics^32,46^. Including soil C stock changes in the total C accounting scheme following reforestation would increase its accuracy^67^. Although, the calculation and verification of the effect of reforestation on soil carbon stocks remains complex and challenging to non-specialists^68^, validated methodologies could improve the attraction of reforestation/afforestation projects on degraded land where potential soil C gain is higher than, for example, that in grassland ecosystems with fertile soils^69–71^.

Overall, because the carbon dioxide released into the atmosphere will, on average, remain there for centuries^72^, the carbon credit sold as an emission permit must ensure that the carbon accounted for is also sequestered for centuries. Our modelling shows that the carbon sequestration claims of reforestation platforms are likely to be unreliable if they do not allow for the time dependency of the carbon capture by planted trees, the risks of tree failure and harvest, and potential changes in soil carbon. Reforestation/afforestation projects obtain massive investments worldwide and are a potential loophole for companies with historically high GHG emissions. Therefore, it is important to emphasise that the carbon capture of reforested/afforested sites relies on the permanence of the tree stand and the end-of-life use of any harvested trees. Accounting for actual carbon stocks in growing forests needs a transparent quantification of the risks, constant monitoring and relevant functional units. There should also be the possibility of withdrawal in case of losses. It is necessary to distinguish between predictions of future carbon sequestration and validated carbon sequestration. While selling future carbon sequestration units can provide reforestation projects with financial inputs to help development and growth, the credits should not be recognised as contributing to a company’s greenhouse gas budget until the carbon has been sequestered and validated. Differentiation between “actual carbon stock increase” and “future carbon credits” should be clarified and marketed differently.

The global warming impact of planting seedlings, which is per se a GHG emitting process, can be lowered by using biochar. Reducing the global warming impact by planting trees is a useful first step, which is context-specific, but validation of the extent and permanence of the future growth is also required.

## Supplementary Information

Supplementary Information.

## Data Availability

All data generated or analysed during this study are included in this published article (and its Supplementary Information files).
