# Supplementary material for: Assessing the carbon capture potential of a reforestation project
Source: Sci Rep. 2021 Oct 7;11:19907. doi: 10.1038/s41598-021-99395-6 (PMC8497602; doi:10.1038/s41598-021-99395-6)
Supplement: Supplementary file 1 — Supplementary Information. [file 41598_2021_99395_MOESM1_ESM.pdf]

Supplementary Information

Assessing the carbon capture potential of a reforestation project

David Lefebvre [a,*], Adrian G. Williams [a], Guy J.D. Kirk [a], Paul, J. Burgess [a], Jeroen Meersmans [b], Miles R. Silman [c,d,e], Francisco Román-Dañobeytia [c,d], Jhon Farfan [c], and Pete Smith [f]

[a] School of Water, Energy and Environment, Cranfield University, Cranfield, Bedfordshire, MK43 0AL, UK

[b] TERRA Teaching and Research Centre, Gembloux Agro-Bio Tech, University of Liège, Gembloux 5030, Belgium

[c] Centro de Innovación Científica Amazónica – CINCIA, Madre de Dios 17001, Peru

[d] Center for Energy, Environment and Sustainability, Wake Forest University, NC 27106, USA

[e] Department of Biology, Wake Forest University, NC 27106, USA

[f] Institute of Biological and Environmental Sciences, University of Aberdeen, 23 St Machar Drive, Aberdeen AB24 3UU, UK

*Correspondence to d.lefebvre@cranfield.ac.uk

1. Meteorological and soil data

Table 1: Latitude, longitude, yearly average temperature and monthly precipitation values from the ClimWat database [1] of the meteorological station considered in this study. These meteorological values were assumed in repetition for the modelling activities.

**Meteorological station**		Puerto Maldonado	
**Altitude**	266 m.a.s.l.		
**Latitude**	-12.63	**Longitude**	-69.2
**Month**	**Average Temperature (°C)**	**Average Precipitation (mm)**	**Evapotranspiration (mm)***
January	26.3	317	143.2
February	25.9	317	120.7
March	25.65	273	125.5
April	25.5	149	116
May	24.45	85	101.7
June	23.25	51	82.2
July	23.1	55	83.6
August	24.25	66	100.3
September	25.8	100	122.8
October	26.3	153	139.3
November	26.65	223	144.1
December	26.35	285	144.9

*Thornthwaite potential evapotranspiration was calculated using the R software package “SPEI” [2]

Table 2: Soil characteristics of the case study plot and the reference forest [3].

**Characteristic**	**Unit**	**Reference Forest**	**Open sandy area (case study)**
pH	/	4.8	5.6
Organic matter	%	3	0.2
CEC	Cmol kg^{-1}	17.4	5.9
Sand content	%	60	91.7
Clay content	%	13.6	5.1
Silt content	%	26.5	3.1
Bulk density	g cm^{-3}	1.11	1.57
Carbon stock (0-20 cm) *	t ha^{-1}	66.63	6.35

* Calculated

2. Nursery

Table 3: Elements needed for the growth of 1000 seedlings in the nursery [4].

Amendment	Weight for 1000 seedlings [kg]
Semi carbonized rice husk	100
Saw dust	75
Poultry manure	150
Cow manure	100
Compost	100
NPK 16-8-12 (Basacote)	3
Fertiliser (Magnocal)	3
Diammonium phosphate	3
NPK 14-16-10 (Yaramilla)	3
Potassium chloride	0.3
Fungicide (Cupravit - copper oxychloride)	0.2
Fungicide (Benzomil - $C_{14}H_{18}N_4O_3$)	0.3

The nursery pays 9 soles per month for the electricity (Jhon Farfan, *pers. com.*). In 2013 the price per kWh in Madre de Dios was 0.44 soles per kWh [5].

3. Carbon modelling data

Table 4: Data for carbon modelling activities.

Parameter	Value	Unit	Note	Reference
CINCIA planting density	1111	Seedling ha^{-1}		3
Biochar yield	30	%		6
soil thickness	20	cm		3
DPM:RPM ratio	0.25	/	For a deciduous or tropical woodland	7
Clay content	5.13	%		3
Degraded soil carbon stock	6.35	t C ha^{-1}	Calculated	3
Adjacent forest soil carbon stock	66.63	t C ha^{-1}	Calculated	3
Time between minimum and maximum litter input	40	years		8
Minimum litter input	1.203	t C ha^{-1}	Inverse RothC	7
Maximum litter input	11.4258	t C ha^{-1}	Inverse RothC	7
Sigmoid equation between minimum and maximum litter input	dI/dt = Min_Litter_Input*I*(1-I/Max_Litter_Input)		Computes a sigmoid between min and max litter input to be used in RothC	

Biochar Carbon content	87.6	%		[9]
Biochar weight considered labile	5	%		[10]
Biochar weight considered recalcitrant	95	%		[10]
Biochar C lost after 100 years	15	%		[6]
Median number of trees in old growth tropical forest	565	/		[11]
Above-ground model	11.47±0.24*sqrt (year)	t C ha^{-1}	For moist forest, non-plantation	[12]
Below-ground model (root to shoot ratio)	0.293±0.0195 - 0.657±0.0978	/	young – old trees	[13]

4. LCA Data

Table 5: Data and emission factors used for the LCA.

Parameter	Value	Unit	Reference	Comment
Distance biomass collection to biochar production plant	15-25	km	[14]	
Distance biochar production plant to reforestation plot	36	km	[14]	
Distance nursery to reforestation plot	128	km	[14]	
Boat fuel consumption to reforestation plot	4.25	litres	(Jhon Farfan, *pers. com.*)	
Weight of seedlings	110	g $seedling^{-1}$	(Jhon Farfan, *pers. com.*)	
GWP - CH_4	28	kg CO_2e kg^{-1}	[15]	
GWP - NO_x	-11	kg CO_2e kg^{-1}	[15]	
GWP - CO	2.65	kg CO_2e kg^{-1}	[15]	
GWP - N_2O	265	kg CO_2e kg^{-1}	[15]	
Density gasoline low sulphur	850	kg m^{-3}	[16]	
Emission factor gasoline	0.88	kg CO_2e kg^{-1}	[17]	Petrol, low-sulfur {RoW}| market for | Alloc Def, S
Emission factor loader operation	3.2	kg CO_2e l of $diesel^{-1}$	[17]	Diesel, combusted in industrial equipment/US
Emission factor electricity	0.392	kg CO_2e kWh^{-1}	[17]	Electricity, low voltage {PE}| market for | Alloc Def, S
Emission factor human labour	0.123	kg CO_2e hour of $work^{-1}$	[18]	Using the purchasing power parity of Peru
Emission factor transportation	0.2628	kg CO_2e tkm^{-1}	[17]	Transport, freight, lorry 7.5-16 metric ton, EURO3 {RoW}| transport, freight, lorry 7.5-16 metric ton, EURO3 | Alloc Def, S – Added 20% to account for rough terrain
Loading operation	0.180 - 0.195	l of diesel tBC^{-1}	[19]	
Emission factor open dump of husks	0.0853	kg CO_2e kg of $husks^{-1}$	[17]	Waste wood, untreated {GLO}| treatment of waste wood, untreated, open dump, very wet infiltration class (1000mm) | Cut-

				off, U (of project Ecoinvent 3 - allocation, cut-off by classification - unit)
Methane production during pyrolysis	30 ± 60	g CH_4 kg biochar^{-1}	[20]	
Nitrogen oxides production during pyrolysis	0.4 ± 0.3	g NO_x kg biochar^{-1}	[20]	
Carbon monoxide production during pyrolysis	54 ± 35.1	g CO kg biochar^{-1}	[20]	
Nursery amendment transportation to city	150	km	Estimated	
Electricity for nursery	0.09	kWh seedling^{-1}	Calculated	
Team number for field work and maintenance	8	people	(Jhon Farfan, *pers. com.*)	
Weight of team member for field work and maintenance	85	kg	Estimated – including gears	
Number of days needed for field work and number of maintenances	3	day ha^{-1}	(Jhon Farfan, *pers. com.*)	
Saw dust	0.0293	kg CO_2e kg^{-1}	[17]	Saw dust, wet, measured as dry mass {GLO}| market for | Alloc Def, S
Poultry manure	0.196	kg CO_2e kg^{-1}	[17]	Poultry manure, fresh {GLO}| nutrient supply from poultry manure, fresh | Conseq, S
Cow manure	0.0388	kg CO_2e kg^{-1}	[17]	Manure, solid, cattle {GLO}| nutrient supply from manure, solid, cattle | Conseq, S
Compost	0.211	kg CO_2e kg^{-1}	[17]	Biowaste {RoW}| treatment of, composting | Alloc Def, S
NPK 16-8-12 (Basacote)	0.903	kg CO_2e kg^{-1}	[17]	NPK compound (NPK 15-15-15), at regional storehouse/RER Mass
Fertiliser (Magnocal)	0.903	kg CO_2e kg^{-1}	[17]	NPK compound (NPK 15-15-15), at regional storehouse/RER Mass
Diammonium phosphate	2.87	kg CO_2e kg^{-1}	[17]	Nitrogen fertiliser, as N {RoW}| diammonium phosphate production | Alloc Def, S
NPK 14-16-10 (Yaramilla)	0.903	kg CO_2e kg^{-1}	[17]	NPK compound (NPK 15-15-15), at regional storehouse/RER Mass
Potassium chloride	0.568	kg CO_2e kg^{-1}	[17]	Potassium chloride, as K2O {GLO}| market for | Alloc Def, S
Fungicide (Cupravit - copper oxychloride)	11	kg CO_2e kg^{-1}	[17]	Pesticide, unspecified {GLO}| market for | Alloc Def, S
Fungicide (Benzomil - $C_{14}H_{18}N_4O_3$)	8.43	kg CO_2e kg^{-1}	[17]	Benzimidazole-compound {GLO}| market for | Alloc Def, S

5. CINCIA's LCA contribution analysis

Table 6: Contribution analysis of the LCA related to the setup of CINCIA's reforestation plot.

Processes	Sub-Process	Values (kg C ha^{-1})	SD (kg C ha^{-1})	Group	Contribution within Group
Biochar plant	Biomass transportation	4.87	0.447	Emission	0.9%
Biochar plant	Loader Operations	1.21	0.0198	Emission	0.2%
Biochar plant	Pyrolysis emissions	298	161	Emission	57.2%
Biochar plant	Biochar Transport	4.92	0.0971	Emission	0.9%
Nursery	Amendments	55.9	16.2	Emission	10.7%
Nursery	Electricity	11.0	/	Emission	2.1%
Nursery	Seedling transport	3.11	0.0380	Emission	0.6%
Field works	On-site fertilizers	25.2	0.0140	Emission	4.8%
Field works	Team Transportation	13.8	0.282	Emission	2.7%
Field works	Nitrous Oxide emissions	76.1	23.3	Emission	14.6%
Field works	Human labour	6.50	/	Emission	1.2%
Field maintenance	Team Transportation	13.8	0.282	Emission	2.7%
Field maintenance	Human labour	6.50	/	Emission	1.2%
Avoided composting	Avoided composting	86.2	/	Emission reduction	8.4%
Biochar in soil	Biochar in soil	942	/	Emission reduction	91.6%

6. Distribution - Monte Carlo

**Methane emissions during pyrolysis**		Unit	Comments
Reported in the LCA	30	kg tBC^{-1}	Literature
Shape	0.25		
Scale	120		
Distribution	Gamma		
N	3		

**Nitrogen oxides emissions during pyrolysis**		Unit	Comments
Reported in the LCA	0.4	kg tBC^{-1}	Literature
Shape	1.78		
Scale	0.225		
Distribution	Gamma		
N	3		

**Carbon monoxide emissions during pyrolysis**		Unit	Comments
Reported in the LCA	54	kg tBC^{-1}	Literature
Shape	2.38		
Scale	22.7		
Distribution	Gamma		
N	3		

**Emission factor direct emission of N_2O-N**		Unit	Comments

Reported in the LCA	0.01	kg kg-N^{-1}	Literature
Min	0.003		
Max	0.3		
Mode	0.01		
Distribution	Triangular		
N	1		

**Emission factor indirect emissions N_2O volatilisation**		Unit	Comments
Reported in the LCA	0.01	kg kg-N^{-1}	Literature
Min	0.002		
Max	0.05		
Mode	0.01		
Distribution	Triangular		
N	1		

**Emission factor indirect emissions N_2O leaching**		Unit	Comments
Reported in the LCA	0.0075	kg kg-N^{-1}	Literature
Min	0.0005		
Max	0.025		
Mode	0.0075		
Distribution	Triangular		
N	1		

**Fraction gasification volatilisation synthetic fertilizer N_2O-N**		Unit	Comments
Reported in the LCA	0.1	/	Literature
Min	0.03		
Max	0.3		
Mode	0.1		
Distribution	Triangular		
N	1		

**Fraction gasification volatilisation organic fertilizer N_2O-N**		Unit	Comments
Reported in the LCA	0.2	/	Literature
Min	0.05		
Max	0.5		
Mode	0.2		
Distribution	Triangular		
N	1		

**Fraction leaching N fertilizer**		Unit	Comments
Reported in the LCA	0.3	/	Literature
Min	0.1		
Max	0.8		
Mode	0.3		
Distribution	Triangular		
N	1		

**Distance between biomass and pyrolysis plant**		Unit	Comments
Reported in the LCA	20	km	
Range	15 – 20 – 25	km	Google Maps

Distribution	Uniform		
N	3		

**Distance transport nursery amendment**		Unit	Comments
Reported in the LCA	150	km	
Range	100 - 200	km	Arbitrary
Distribution	Uniform		
N	2		

**Loader operation**		Unit	Comments
Reported in the LCA	0.188	l diesel tBC^{-1}	
Range	0.18 – 0.195	l diesel tBC^{-1}	Literature
Distribution	Uniform		
N	2		

**Emissions factor truck transportation**		Unit	Comments
Reported in the LCA	0.2409	kg CO_2 tkm^{-1}	
Range	0.219 - 02628	kg CO_2 tkm^{-1}	EcoInvent and additional 20% for rough terrain
Distribution	Uniform		
N	2		

**Emission factor carbon monoxide**		Unit	Comments
Reported in the LCA	2.65	kg CO_2 kg^{-1}	
Range	2 – 3.3	kg CO_2 kg^{-1}	Literature
Distribution	Uniform		
N	2		

7 References

1. FAO. CLIMWAT 2.0 for CROPWAT. (2006).

2. Beguería, S. & Vicente-Serrano, S. M. SPEI: Calculation of the Standardised Precipitation-Evapotranspiration Index. (2017).

3. Román-Dañobeytia, F. *et al.* Survival and early growth of 51 tropical tree species in areas degraded by artisanal gold mining in the Peruvian Amazon. *Ecol. Eng.* **159**, 106097 (2021).

4. Farfan, J. F. *Produccion de Plantones en el Vivero Tecnificado de Mazuko Para Recuperar Suelos Degradado por Mineria Aluvial en Madre de Dios*. (2020).

5. MINEM. *Capítulo 5: Por qué cuesta la energía y cómo calculamos nuestra factura*. *Guía Inicial de la Energía* http://www.minem.gob.pe/giee/pdf/GUIA_INICIAL_PRIMARIA_CAP5.pdf (2013).

6. IPCC. Appendix 4: Method for Estimating the Change in Mineral Soil Organic Carbon Stocks from Biochar Amendments. in *2019 Refinement to the 2006 IPCC Guidelines for National*

Greenhouse Gas Inventories 2–6 (2019).

7. Coleman, K. & Jenkinson, D. S. *RothC - A model for the turnover of carbon in soil Model - Model description and users guide*. https://www.rothamsted.ac.uk/sites/default/files/RothC_guide_WIN.pdf (2014).

8. Jones, I. L. *et al.* Above- and belowground carbon stocks are decoupled in secondary tropical forests and are positively related to forest age and soil nutrients respectively. *Sci. Total Environ.* **697**, 133987 (2019).

9. Lefebvre, D. *et al.* Biochar Effects on Two Tropical Tree Species and Its Potential as a Tool for Reforestation. *Forests* **10**, 678 (2019).

10. Calvelo Pereira, R. *et al.* Contribution to characterisation of biochar to estimate the labile fraction of carbon. *Org. Geochem.* **42**, 1331–1342 (2011).

11. ter Steege, H. *et al.* Hyperdominance in the Amazonian Tree Flora. *Science (80-.).* **342**, 1243092–1243092 (2013).

12. Busch, J. *et al.* Potential for low-cost carbon dioxide removal through tropical reforestation. *Nat. Clim. Chang.* **9**, 463–466 (2019).

13. Waring, B. G. & Powers, J. S. Overlooking what is underground: Root:shoot ratios and coarse root allometric equations for tropical forests. *For. Ecol. Manage.* **385**, 10–15 (2017).

14. Google. Google Earth Pro. https://www.google.com/earth/download/gep/agree.html (2018).

15. Myhre, G. *et al.* Anthropogenic and Natural Radiative Forcing. in *Climate Change 2013: The Physical Science Basis. Contribution of Working Group I to the Fifth Assessment Report of the Intergovernmental Panel on Climate Change* (eds. Jacob, D., Ravishankara, A. R. & Shine, K.) (Cambridge University Press, 2013). doi:10.3390/jmse6040146.

16. Shell. Marine Fuels. *Shell Trading and Supply* 1–2 https://www.shell.com/business-customers/marine/fuel/ulsfo.html (2014).

17. Wernet, G. *et al.* The ecoinvent database version 3 (part I): overview and methodology. *Int. J. Life Cycle Assess.* **21**, 1218–1230 (2016).

18. Rugani, B., Panasiuk, D. & Benetto, E. An input-output based framework to evaluate human labour in life cycle assessment. *Int. J. Life Cycle Assess.* **17**, 795–812 (2012).

19. Rosado, L. P., Vitale, P., Penteado, C. S. G. & Arena, U. Life cycle assessment of natural and mixed recycled aggregate production in Brazil. *J. Clean. Prod.* **151**, 634–642 (2017).

20. IPCC. Volume 2, Chapter 4: Fugitive Emissions - 2019 Refinement to the 2006 IPCC

Guidelines for National Greenhouse Gas Inventories. *IPCC Guidel. Natl. Greenh. Gas Invent.* **2**, 78 (2019).
